# The Effectiveness of Ketamine Versus Opioids in Patients With Acute Pain in the Emergency Department: A Systematic Review and Meta-Analysis

**DOI:** 10.7759/cureus.36250

**Published:** 2023-03-16

**Authors:** Bsaim A Altirkistani, Alaa A Ashqar, Dena M Bahathiq, Suaad M Bougis, Ammar M Aljabri, Sawsan Hanafi

**Affiliations:** 1 College of Medicine, King Saud Bin Abdulaziz University for Health Sciences, Jeddah, SAU; 2 Department of Emergency Medicine, King Abdulaziz Medical City, Jeddah, SAU

**Keywords:** pain score, emergency department, morphine, ketamine, acute pain

## Abstract

Opioids are the mainstay of treatment for acute pain in the emergency department. However, its misuse led to the investigation of alternative effective analgesic options for acute pain complaints such as ketamine. Therefore, this systematic review and meta-analysis aimed to determine the effectiveness of ketamine in comparison to opioids in the management of acute pain. This was a systematic review and meta-analysis of randomized controlled trials comparing ketamine to opioids for the relief of acute pain in the ED. Eligible studies were identified by searching the following electronic databases: Medline, Embase, and Central. Studies utilizing either the visual analog scale (VAS) or the numeric rating scale (NRS) for pain scoring in ketamine vs opioids were included. The revised Cochrane risk-of-bias tool for randomized trials was utilized. A random-effects model was performed, and all outcomes were pooled by the inverse variance weighting method. The total number of studies that met the criteria of systematic reviews was nine of which seven of them were included in the meta-analysis with 789 participants. The overall effect of NRS trials was the standardized mean difference (SMD) = -0.07, 95% confidence interval (CI) -0.31 to 0.17, P-value = 0.56, I^2^ =85%. While VAS trials showed an overall effect of SMD = -0.02, 95% CI -0.22 to 0.18, P = 0.84, I^2 ^= 59%). Moreover, higher adverse events were reported in opioids; however, this was not statistically significant (SMD = 1.23, 95% CI 0.93-1.64, P = 0.15, I2 =38%). Ketamine for immediate pain relief at 15 minutes could be an effective alternative to opioids, but its overall effect in comparison to opioids for improving the pain has not shown a statistically significant difference. There was high heterogeneity in the included studies; thus, a sub-group analysis was performed.

## Introduction and background

Acute pain is one of the most common presentations in the emergency department. It represents more than half of the emergency department visits [1]. The pain assessment can be determined by different types of scales with the visual analog scale (VAS) and numerical rating scale (NRS) being the most commonly used, as they are sensitive to the subjective feeling of patients’ pain [1]. Furthermore, appropriate and prompt management of such complaints is crucial. Despite opioids being the most used medication for acute pain, they are not advisable to be given to all patients presenting with acute pain due to risks of hypotension and hypoxemia.

Safer alternative options could provide comparable pain control with a better side effect profile [2,3]. Ketamine, for instance, is an opioid alternative [3]. It is an N-methyl-D-aspartate receptor antagonist and has been used widely for procedural sedations and intubations. High doses of ketamine may activate other receptors and result in undesirable adverse effects such as short-term hallucinations, night-time dreams, psychosis, dizziness, and blurry vision. Nevertheless, to lessen the experience of such symptoms, it is proper to use a low dose of ketamine, which also has been shown to possess an analgesic effect for pain. If ketamine was given, it can lead to a decrease in the overconsumption of opioids alongside to effectively manage acute pain [2,4].

Moreover, a conducted systematic review and meta-analysis (SRMA) showed that ketamine was non-inferior to morphine in the control of acute pain [3]. Also, it was reported that ketamine efficacy varies with the site of pain; nevertheless, a low dose of ketamine showed to be an alternative to opioid use, and there were no reported side effects [4]. Therefore, this systematic review and meta-analysis aimed to determine whether ketamine administration is more effective than opioids in acute pain management.

## Review

Methods

This systematic review and meta-analysis was conducted in accordance with Preferred Reporting Items for Systematic Reviews and Meta-Analyses (PRISMA) reporting guidelines and checklists. This systematic review was not registered in PROSPERO due to a technical issue.

Search strategy

**Figure 1:**
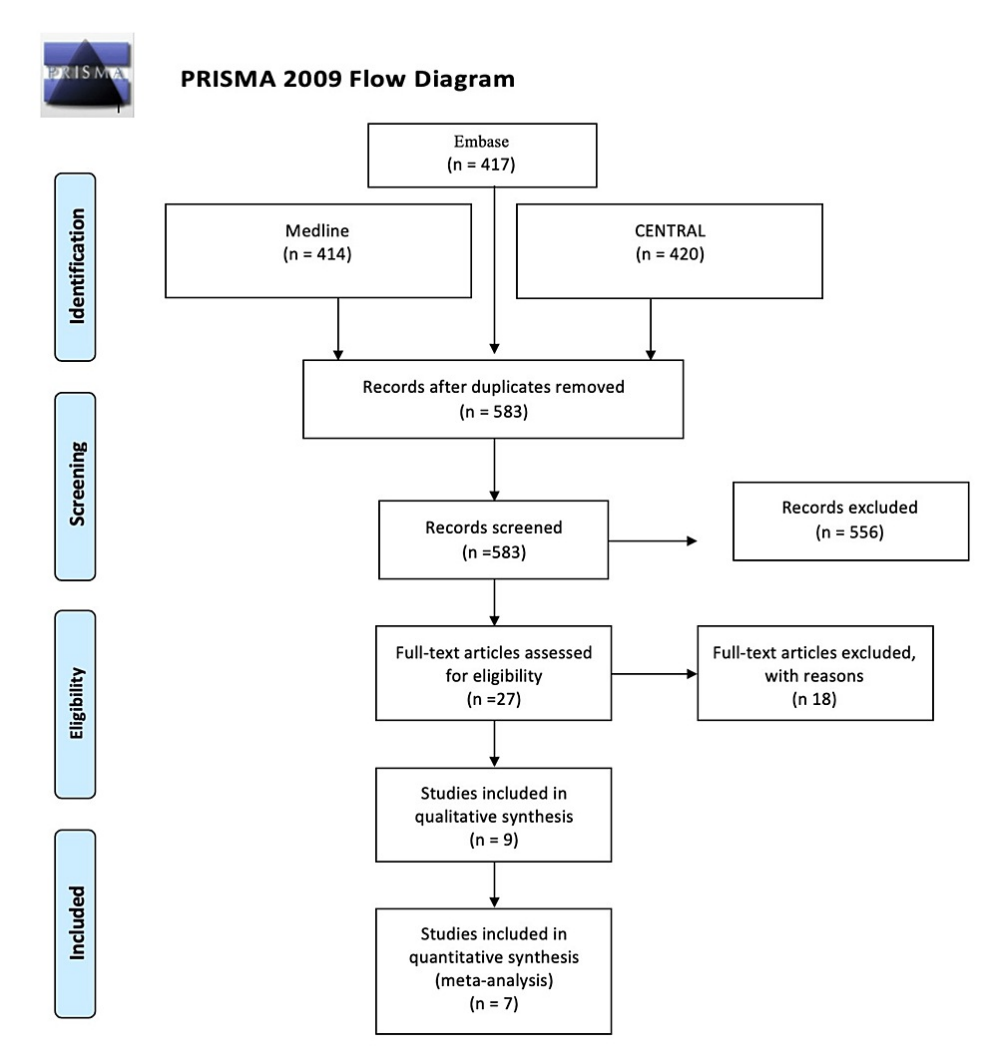
PRISMA flow chart summarizing the literature search PRISMA: Preferred Reporting Items for Systematic Reviews and Meta-Analyses

The electronic databases Medline, Embase, and Central were used to reach the clinical trials that discussed our topic of interest. The keywords used for the search were ketamine, acute pain, opioids, visual analog score, and decrease pain (Figure 1).

Study selection

Two independent researchers reviewed the studies initially based on titles, then by screening abstracts, and lastly full paper screening to determine whether inclusion and exclusion criteria were met or not. Any disagreements were resolved by discussion and justification between the two reviewers. An additional independent reviewer was consulted when any disagreements could not be resolved. The inclusion criteria were as follows: (1) randomized controlled trials (RCT) addressing the effect of ketamine use on acute pain management vs opioids use in the emergency department; (2) studies that investigated acute pain among patients ages 18 years and old; and (3) studies that either they used the VAS or NRS for pain assessment. While the use of other pain scales or the presence of a placebo in the comparison group were the exclusion criteria.

Data extraction

The data were extracted independently by two researchers from the full-text reports and supplemental materials. The following information was entered into a data extraction form: the number of patients in each group, mean age, gender, cause of emergency department visit, and the type of pain assessment score used. Also, the baseline pain score prior to the administration of the medication and the score after which the dose was given. Furthermore, the routes of administration and side effects resulting from the administered medication were extracted. Disagreements were resolved through the discussion among the researchers.

Risk of bias assessment

**Figure 2:**
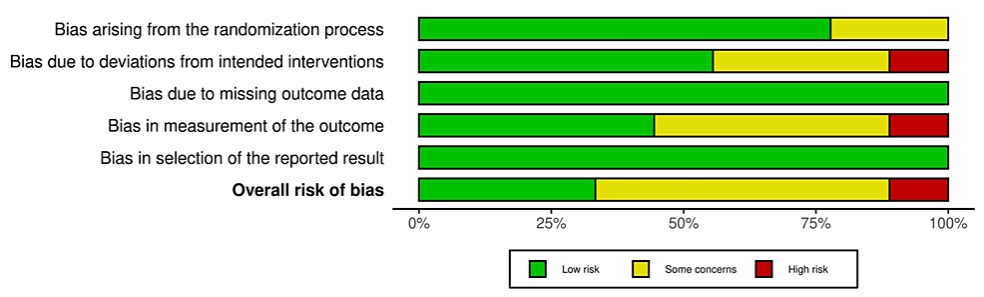
Risk of bias

**Figure 3:**
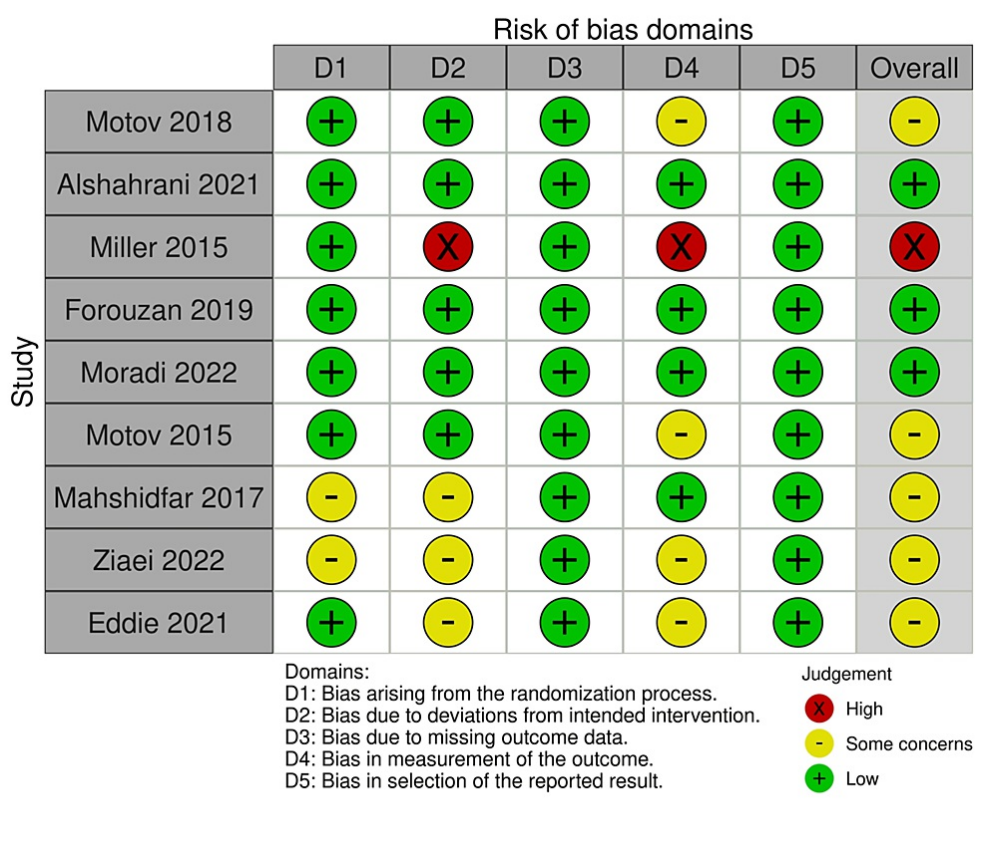
Risk of bias domains Miller 2015 [5], Motov 2015 [6], Motov 2018 [7], Mahshidfar 2017 [8], Eddie 2021 [9], Ziaei 2022 [10], Forouzan 2019 [11], Moradi 2022 [12], Alshahrani 2021 [13]

The risk of bias for the included RCTs was independently assessed by two reviewers using the Revised Cochrane risk-of-bias tool for randomized trials. Low, some concern, and high risk were determined by assessing the following domains: randomization process, allocation concealment, blinding of participants and personnel, blinding of outcome assessors, incomplete outcome data, selective reporting, and other biases. Disagreements were resolved through the discussion among the researchers. Refer to Figure 2 and Figure 3.

Outcome measures

The primary outcome of the current review was to compare the efficacy of ketamine use to opioids in acute pain reduction in patients presented to the emergency department using NRS or VAS. Values were sub-grouped according to the time of assessment for NRS studies (15, 30, 60, 90, and 120 minutes) while it was 10, 20, 30, and 60 minutes for VAS studies. The secondary outcome was to estimate and compare the frequency of adverse events among the included drugs.

Statistical analysis

The meta-analysis was conducted using Review Manager (RevMan5) version 5.3 software (Cochrane Collaboration, London, United Kingdom). A random-effects model was applied, and all outcomes were pooled by the inverse variance weighting method. Continuous outcomes were represented using Standardized Mean Difference (SMD) with a confidence interval (CI) of 95%. Risk ratio (RR) was used for dichotomous variables. A p-value<0.05 was considered significant. The I^2^ statistic along with the Chi-squared test was used to test for statistical heterogeneity, where an I^2^ value greater than 50% was considered significant heterogeneity.

Results

The total number of patients in the seven studies in a meta-analysis of pain scores was 789; of them, 398 patients received ketamine and 391 patients received opioids. Out of these studies, five studies used the NRS pain score [5-9] while two studies used the VAS pain score [10,11]. These pain score measurements were compared, and the result was considered the primary outcome of the study (Table 1).

**Table 1:** Studies included in the systematic review

Study ID	Year	Sample Size Ketamine/Opioids	Medication Route/Dose: Ketamine vs. Opioids		Pain Scale
Miller [5]	2015	24/21	IV/0.3 mg/kg	IV/0.1 mg/kg	NRS
Motov [6]	2015	45/45	IV/0.3 mg/kg	IV/0.1 mg/kg	NRS
Motov [7]	2018	30/30	IV/0.3 mg/kg	IV/0.1 mg/kg	NRS
Mahshidfar [8]	2017	150/150	IV/0.2 mg/kg	IV/0.1 mg/kg	NRS
Eddie [9]	2021	31/27	IV/0.3 mg/kg	IV/0.1 mg/kg	NRS
Ziaei [10]	2022	50/50	IN 1.5 mg/kg	IV/0.1 mg/kg	VAS
Forouzan [11]	2019	68/68	IV/0.3 mg/kg	IV/0.1 mg/kg	VAS
Moradi [12]	2022	100/100	Haloperidol 2.5 mg/kg ketamine 0.3 mg/kg	IV/1 mg/kg	NRS
Alshahrani [13]	2021	138/140	IV/0.3 mg/kg	IV/0.1 mg/kg	NRS

Primary outcomes

**Figure 4:**
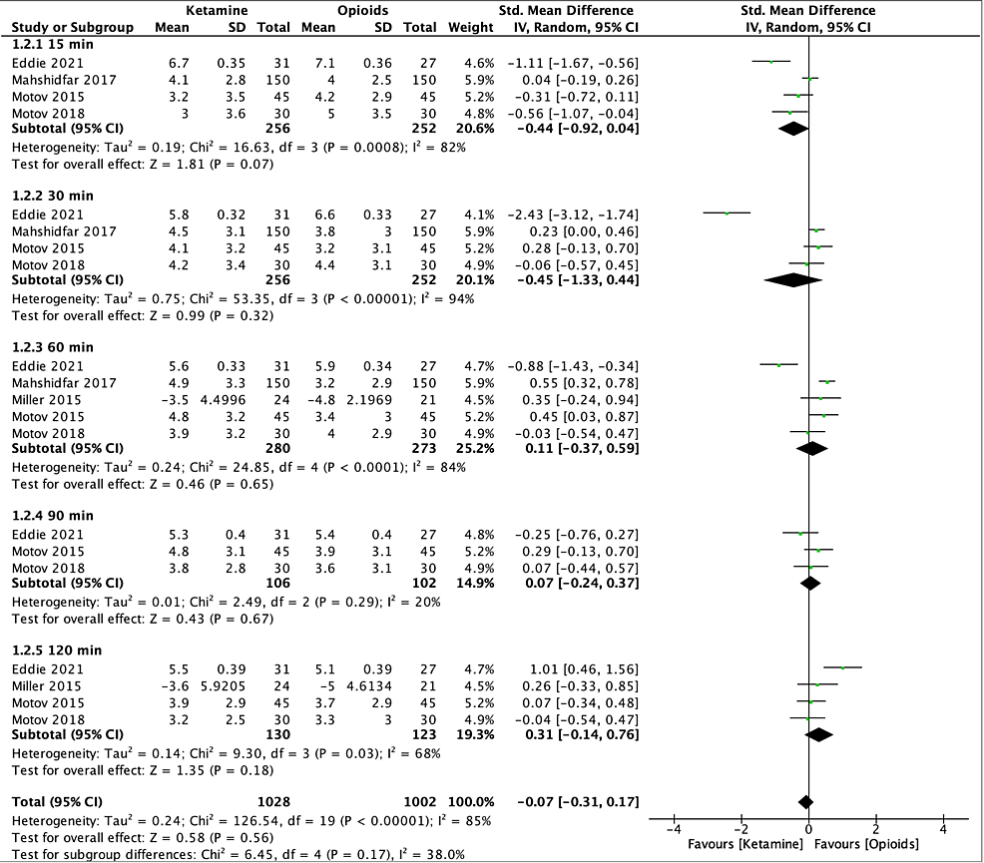
Forest plot of NRS pain scores Miller 2015 [5], Motov 2015 [6], Motov 2018 [7], Mahshidfar 2017 [8], Eddie 2021 [9] NRS: numerical rating scale

The total number of patients whose pain was recorded by the NRS score was 553. The overall effect of studies that utilized the NRS pain score was SMD = -0.07, 95% CI -0.31 to 0.17, P = 0.56, I^2^ = 85%). However, due to high heterogenicity, a sub-group analysis was performed for the NRS pain score trials, which were classified based on different times at which the pain scores were recorded, that is at 15, 30, 60, 90, and 120 minutes. Overall, NRS pain score subgroups showed a non-statistically significant difference (P=0.17) for ketamine to opioids. Refer to Figure 4 for the results of the subgroup analysis.

**Figure 5:**
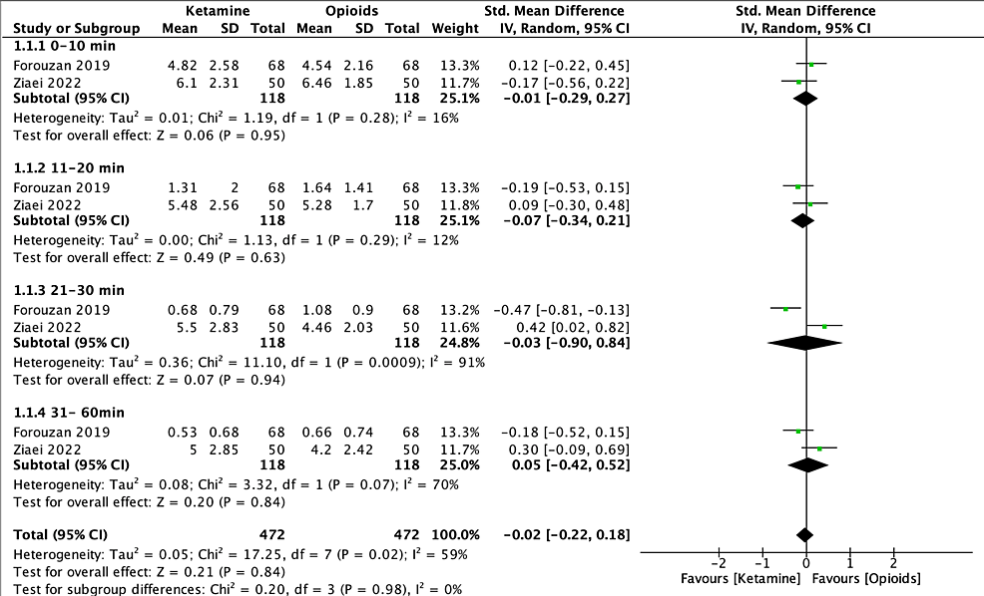
Forest plot of VAS pain scores Ziaei 2022 [10], Forouzan 2019 [11] VAS: visual analog scale

A total of 236 patients had their pain recorded according to the VAS score. They reported an overall effect of SMD = -0.02, 95% CI -0.22 to 0.18, P = 0.84, I^2^=59%. Sub-group analysis was performed because of the high heterogenicity between score trials. Therefore, they have been presented and classified based on different times of pain recording. Nevertheless, this sub-group analysis showed that there was no statistically significant difference (P=0.98) between ketamine and opioids. Refer to Figure 5 for the results of the subgroup analysis.

Secondary outcomes

**Figure 6:**
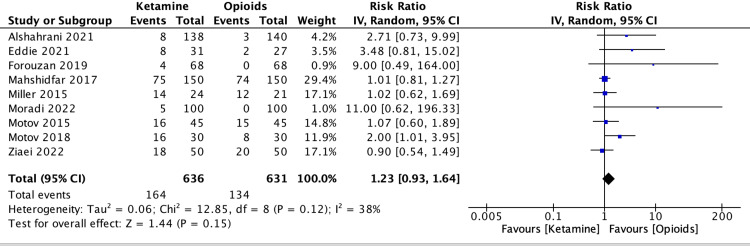
Forest plot of adverse events Miller 2015 [5], Motov 2015 [6], Motov 2018 [7], Mahshidfar 2017 [8], Eddie 2021 [9], Ziaei 2022 [10], Forouzan 2019 [11], Moradi 2022 [12], Alshahrani 2021 [13]

Regarding adverse events, the total number of patients in nine studies who reported adverse events was 164 among ketamine and 134 among opioids. A meta-analysis revealed that opioids were superior to ketamine in the reported adverse events; nevertheless, this was not statistically significant (SMD = 1.23, 95% CI 0.93-1.64, P = 0.15, I^2^ = 38%) (Figure 6).

Discussion

Ketamine is considered to be an anesthetic agent that has been widely utilized for procedural purposes in emergency departments [14,15]. Various studies have investigated its use as an alternative analgesic to opioids due to its effectiveness and greater safety in managing acute pain [3-4,16-18]. Even though opioids are well-known analgesic medications that work to relieve acute pain, their increased correlation with death that result from overdoes and abuse contributes to the importance of further investigating other safer alternatives [19-21].

Miller et al. included 45 patients and reported that ketamine was not superior to morphine in NRS pain score change in which the maximum reduction of the NRS pain score in the ketamine group and in the morphine group was 4.9 vs. 5, respectively. Also, 58% of the ketamine arm vs. 57% of the morphine arm experienced adverse effects [5]. While, Motov 2015 et al. reported that complete resolution pain was seen in the ketamine group specifically at 15 minutes, but no difference was observed at 30 minutes. However, a statistically significant increase in adverse events was documented in the ketamine recipients’ group [6]. Similarly, Motov 2018 et al. reported that at 15 minutes, the ketamine group demonstrated greater pain relief by 2 points while no statistically significant difference was found in pain score at 30-120 minutes between the ketamine and morphine groups. Moreover, a higher rate of adverse events was observed among the ketamine group [7]. Also, Mahshidfar et al. reported that a significant pain score reduction was observed for ketamine morphine groups specifically in the early minutes of dose administration compared to baseline scores. Complications were less in the ketamine group as compared to morphine [8]. In addition, Eddie et al. reported that although the ketamine group had a higher rate of dizziness, ketamine provided a greater antalgics effect for long bone fracture pain compared to morphine over 15 minutes of dose administration [9]. Moreover, Ziaei et al. showed that intravenous morphine was considered faster to reduce renal colic pain than ketamine; nevertheless, prolonged analgesic effects and longer pain control were seen in the ketamine group [10]. However, Forouzan et al. reported that there was no statistically significant difference between ketamine and morphine regarding pain reduction scores at all points except at 30 minutes [11]. Furthermore, Moradi et al. reported that ketamine was effective in controlling acute pain in comparison to fentanyl alongside its greater sedative power, which could contribute to recommending its use over opioids. Also, if ketamine was given with haloperidol, agitation as a side effect of ketamine can be controlled [12]. Lastly, Alsharani et al. reported that the use of ketamine reduced the cumulative dose of opioids to control the pain of a vasoocclusive crisis [13].

In the present SRMA, studies, which utilized NRS revealed that at 15 minutes after medication administration, reduction of pain with ketamine was not found to be inferior to opioids; nevertheless, the difference did not reach a statistical significance. This could be explained by the effectiveness of ketamine to work earlier upon dose administration. Overall, regardless of which time the pain was recorded, ketamine was not found to be superior to opioids. Similarly, RCTs that used VAS scores have also revealed that ketamine was not superior to opioids with a non-statistically significant difference. However, the pooled analysis of Karlow et al. reported that ketamine was, in fact, statistically non-inferior to morphine in reducing pain [3].

The current SRMA revealed that opioids were superior to ketamine in terms of the reported adverse events. The difference, however, was not statistically significant. Previously reported systematic reviews showed that ketamine did not express any life-threatening events [3,16,17]. Higher rates of neurological and psychiatric adverse events were seen in the ketamine group while cardiovascular events were higher among the opioids group, as Lee and Lee reported [4].

## Conclusions

Ketamine for immediate pain relief at 15 minutes could be an effective alternative to opioids, but its overall effect in comparison to opioids for improving the pain has not shown a statistically significant difference. There was high heterogeneity in the included studies; thus, a sub-group analysis was performed. There were also some limitations to the current systematic review and meta-analysis. There was high heterogeneity in the included studies; thus, a sub-group analysis was performed. In addition, we attempted to contact the corresponding author of one of the included trials in order to retrieve certain information that was not available in the original article. However, no response was received; thus, the study was not included in the meta-analysis.
